# Which data subset should be augmented for deep learning? a simulation study using urothelial cell carcinoma histopathology images

**DOI:** 10.1186/s12859-023-05199-y

**Published:** 2023-03-03

**Authors:** Yusra A. Ameen, Dalia M. Badary, Ahmad Elbadry I. Abonnoor, Khaled F. Hussain, Adel A. Sewisy

**Affiliations:** 1 Department of Computer Science, Faculty of Computers and Information, Assiut University, Asyut, Egypt; 2 Department of Pathology, Faculty of Medicine, Assiut University, Asyut, Egypt; 3 Urology and Nephrology Hospital, Faculty of Medicine, Assiut University, Asyut, Egypt

**Keywords:** Convolutional neural network, Data augmentation, Deep learning, Histopathology, Urothelial cell carcinoma

## Abstract

**Background:**

Applying deep learning to digital histopathology is hindered by the scarcity of manually annotated datasets. While data augmentation can ameliorate this obstacle, its methods are far from standardized. Our aim was to systematically explore the effects of skipping data augmentation; applying data augmentation to different subsets of the whole dataset (training set, validation set, test set, two of them, or all of them); and applying data augmentation at different time points (before, during, or after dividing the dataset into three subsets). Different combinations of the above possibilities resulted in 11 ways to apply augmentation. The literature contains no such comprehensive systematic comparison of these augmentation ways.

**Results:**

Non-overlapping photographs of all tissues on 90 hematoxylin-and-eosin-stained urinary bladder slides were obtained. Then, they were manually classified as either inflammation (5948 images), urothelial cell carcinoma (5811 images), or invalid (3132 images; excluded). If done, augmentation was eight-fold by flipping and rotation. Four convolutional neural networks (Inception-v3, ResNet-101, GoogLeNet, and SqueezeNet), pre-trained on the ImageNet dataset, were fine-tuned to binary classify images of our dataset. This task was the benchmark for our experiments. Model testing performance was evaluated using accuracy, sensitivity, specificity, and area under the receiver operating characteristic curve. Model validation accuracy was also estimated. The best testing performance was achieved when augmentation was done to the remaining data after test-set separation, but before division into training and validation sets. This leaked information between the training and the validation sets, as evidenced by the optimistic validation accuracy. However, this leakage did not cause the validation set to malfunction. Augmentation before test-set separation led to optimistic results. Test-set augmentation yielded more accurate evaluation metrics with less uncertainty. Inception-v3 had the best overall testing performance.

**Conclusions:**

In digital histopathology, augmentation should include both the test set (after its allocation), and the remaining combined training/validation set (before being split into separate training and validation sets). Future research should try to generalize our results.

**Supplementary Information:**

The online version contains supplementary material available at 10.1186/s12859-023-05199-y.

## Background

Machine learning, a major branch of artificial intelligence, comprises algorithms that can make predictions after being trained on prior examples. Deep learning, a subset of machine learning, consists of a more recent and more sophisticated category of these algorithms. Deep learning includes, but is not limited to, convolutional neural networks (CNNs), which are capable of directly learning from image datasets [1, 2]. This opened the door for a myriad of applications in medical image analysis [3, 4]. In digital pathology, these applications encompass low-level tasks such as nuclei segmentation, mitosis detection, and gland segmentation; standard applications such as tumor detection, subtyping, grading, and staging; and advanced inferences that cannot be reliably done by human experts such as prediction of survival, recurrence, treatment response, and mutations [5–7].

Before building a CNN model, the convention is to divide the available dataset into three subsets. The test set is first put aside till the final model is built. The remainder of the dataset is divided into two subsets to build the model: the training set and the validation set. Training is just tuning the CNN parameters to approach the target model. A training ‘epoch’ ends when the CNN has seen all of the images in the training set, then the validation set is used to measure the improvement in the model performance. Thereafter, the CNN passes through a new training epoch followed by validation, and so on and so forth. This training/validation cycle repeats until the validation results indicate that the model can barely be further improved. If too many training epochs are run, the CNN will ‘overfit’ the training set. Overfitting renders the model unable to generalize accurately when tested on external data. As such, validation prevents overfitting. Since the test set was not involved in the model building process by any means, it can now be used to evaluate the final model without bias [8, 9].

Nonetheless, histopathological image analysis by deep learning is still not clinically adopted on a wide scale. One reason for this is the inherent ‘black box’ nature of deep learning models. In clinical practice, it is extremely useful to know which pathological features were used by the model to reach its conclusions [6, 10]. Fortunately, substantial progress has been made to enhance the explainability of intelligent system recommendations in general [11, 12], and digital pathology is not an exception [13–15]. The other key challenge that is facing the clinical application of deep learning to histopathology is the scarcity of high-quality manually annotated reference datasets [10, 16, 17]. Even worse, most of the available whole-slide images are labeled at the case level, as opposed to the much more useful patch-level or pixel-level labeling [10, 17]. An abundance of these hand-annotated images is typically needed, not only for developing successful deep learning models, but also for externally validating them. Unfortunately, building such large datasets is greatly laborious and time-consuming [10, 16–18]. Several strategies have been suggested to ameliorate this problem, such as transfer learning [10, 17–19], immunohistochemical markers [18], semi-supervised learning [10, 17, 18, 20], multiple-instance learning [10, 17, 18], and artificial-intelligence-based labeling [21]. However, data augmentation [18, 22–24] remains one of the most prominent strategies in this respect. Dataset augmentation entails increasing the number of images in the dataset either by introducing certain modifications to the original images or by creating synthetic images. Modifying the original images must not alter the features on which the classification is based, but still make the images apparently different. Example modifications include geometric and color transformations and random erasing [25].

While data augmentation is a very promising approach to compensate for histopathological data deficiency, we believe that its full potential has yet to be exploited. Our extensive literature review (see related work below) revealed that in many studies data augmentation was unused, inefficiently used, and/or ambiguously reported. There is a lack of standardization owing to the plethora of augmentation techniques, the great variability of problems where deep learning is applicable, and the many unanswered research questions pertinent to data augmentation.

The motivation of our work was to answer one of these basic questions: holding all other variables constant, which data subset should be augmented to achieve the best possible model performance? Confining our scope to histopathology images, we explored the effects of applying geometric-transformation-based augmentation to different dataset subsets and at different time points relative to dataset division. According to our review of the literature, these augmentation ways have never been compared via a comprehensive systematic approach.

For each of these augmentation ways, we evaluated the ability of four pre-trained CNNs to discriminate between urothelial cell carcinoma (UCC) and inflammation of the urinary bladder. This simple classification task was used as a benchmark for our experiments to reduce the confounding factors to a minimum. Urinary bladder cancer was a suitable choice for two reasons. First, although this disease ranks tenth in worldwide cancer incidence [26], it is underrepresented in digital pathology deep learning studies [5–7, 17, 27, 28]. Second, in a recent study comparing 19 cancer types, bladder cancer was the second easiest-to-classify tissue, the first being breast cancer [29]. This result suggests that these tumors are canonical for studies like ours.

## Methods

**Fig. 1:**
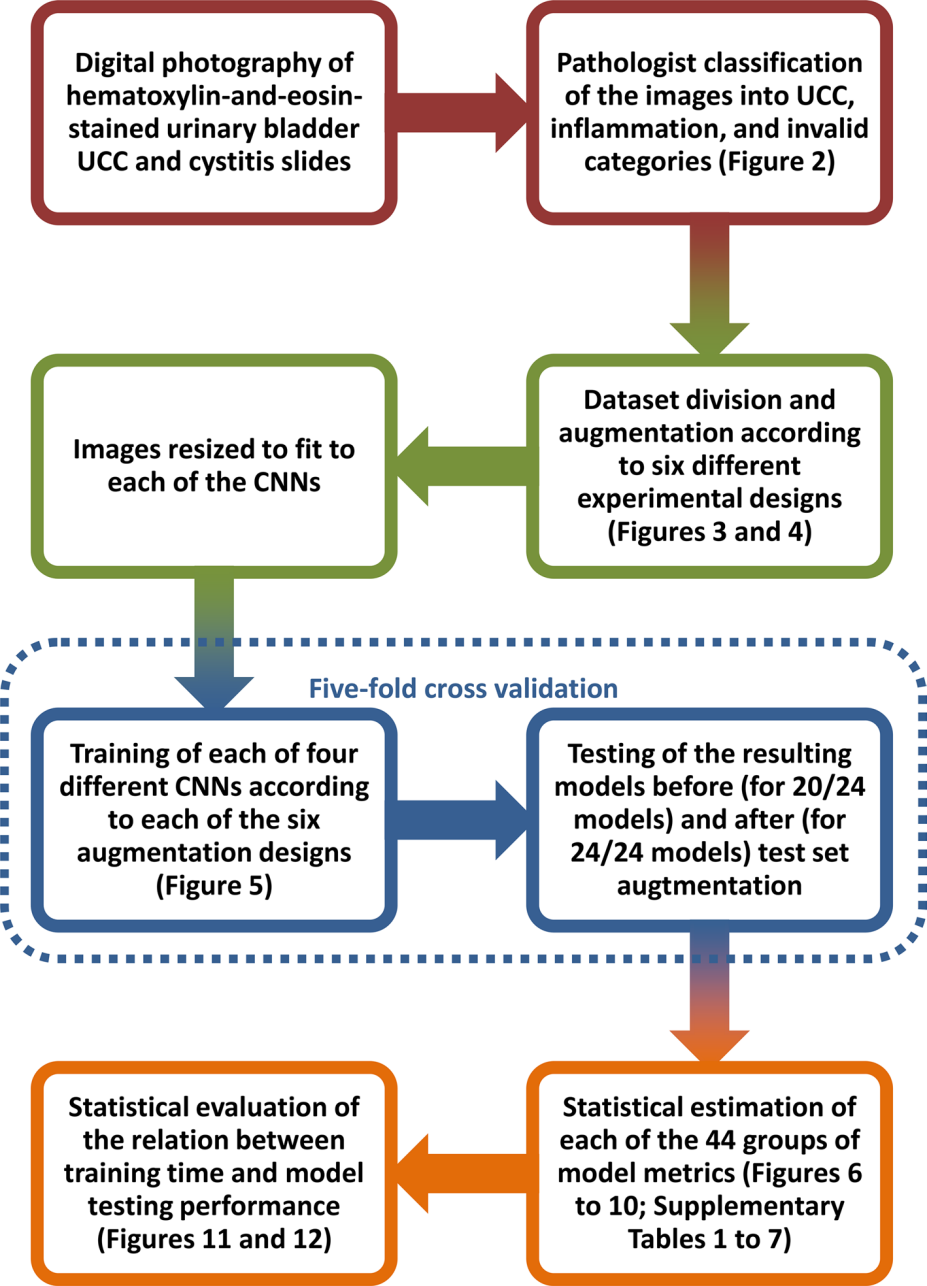
Overall workflow of the study. Each color-coded pair of successive steps represents a different phase, namely, dataset building, dataset preprocessing, model building and testing, and statistical analysis. Note that five-fold cross validation was applied to model building and testing. UCC = urothelial cell carcinoma; CNNs = convolutional neural networks

The overall workflow of the methods comprised dataset building, dataset preprocessing, model building, model testing, and statistical analysis. These stages are overviewed in Fig. 1 and detailed in the next paragraphs.

### Dataset building

The dataset source was 90 formalin-fixed paraffin-embedded hematoxylin-and-eosin-stained histopathology slides with 4-μm-thick sections of urinary bladder lesions that were either cystitis (43 slides) or UCC (47 slides). Slides were obtained from 74 specimens from the Departments of Pathology of both of the Faculty of Medicine and the Cancer Institute in our university. Approval of the Institutional Review Board to publish the dataset was obtained under the number 17300658.

Slides were photographed using an Olympus® E-330 digital camera mounted on an Olympus® CX31 light microscope by an Olympus® E330-ADU1.2X adapter. Magnification of the microscope was set to 20 × . Certain camera settings were adjusted before photographing. The shutter speed, aperture value, International Organization for Standardization (ISO) sensitivity to light, and white balance were set automatically. Exposure compensation value, which controls the brightness, was set to + 1.0. Images were set to have a resolution of 3136 × 2352 pixels, a Joint Photographic Experts Group (JPEG) format, and a 1:2.7 compression rate. Non-overlapping photographs of all available tissue areas on each slide were systematically obtained.

**Fig. 2:**
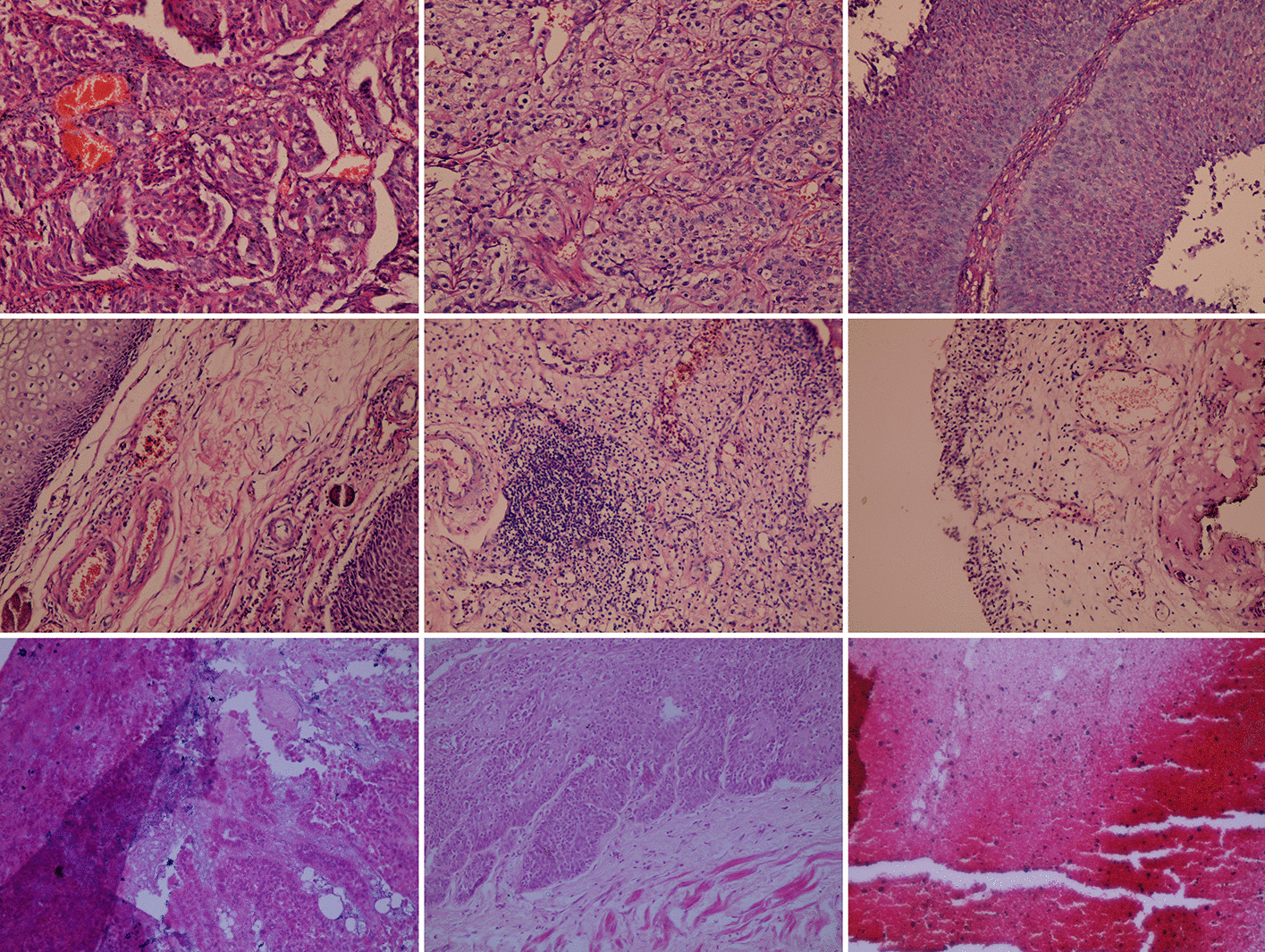
Examples from the histopathology image dataset. Example images from the urothelial cell carcinoma (upper row), the inflammation (middle row), and the invalid (lower row) categories as classified manually by the pathologist in the study

Regardless of the slide-level diagnoses, the pathologist in our group manually classified all of the obtained images into three categories: inflammation, UCC, and invalid (Fig. 2). An image-level (also known as patch-level) diagnosis of inflammation was based on the presence of inflammatory cell infiltrate in the form of lymphocytes, plasma cells, eosinophils, and/or polymorphs, in the absence of any malignant cells. An image-level diagnosis of UCC was based on the presence of malignant urothelial cells showing features of anaplasia in the form of pleomorphism, hyperchromatism, increased nuclear-cytoplasmic ratio, and increased mitotic figures. These malignant cells may be arranged in papillae, sheets, or groups. They may also be present as single cells. An image was considered invalid when it contained no sufficient criteria to be included in one of the other two categories, even if it only contained normal urinary bladder tissue. Also, an image was considered invalid if it contained tissues that were processed too badly to be diagnosed. The pathologist’s classification resulted in a total of 5948 inflammation images, 5811 UCC images, and 3132 invalid images. Invalid images were excluded. The final dataset was published at [30]. The pathologist’s labeling of these images was considered the ground truth in the subsequent steps of the study.

### Dataset preprocessing

Instead of wasting a part of the precious dataset for testing, k-fold cross-validation makes use of the whole dataset. First, the dataset is divided into k equal parts. Next, each part is used as a test set for a model built using the combined remaining k-1 parts. This results in a total of k models. The individual evaluation metrics of these k models are combined in a statistically sound way to reach less biased overall metrics. In stratified k-fold cross-validation, the proportion of classes in the whole dataset is preserved while dividing the dataset into k parts [8, 31, 32]. In our work, we used stratified five-fold cross-validation. In each fold, the four parts used for model building were first combined, shuffled, and then divided into a training set and a validation set in a ratio of 3:1, respectively. The proportion of classes was also preserved during the latter division.

As such, the dataset is divided before deep learning into a training subset, a validation subset, and a test subset in the ratio of 3:1:1, respectively. This division is repeated five times per deep-learning experiment, using five non-intersecting test subsets, to perform five-fold cross-validation.

Data augmentation can be done before, during, or after this division, or it may be skipped altogether. Furthermore, augmentation is not necessarily applied to all three subsets, but may be applied to just one or two of them. Starting with the whole dataset, different combinations of these possibilities resulted in six different ways to apply data augmentation before model building, five of which could be tested both before and after test-set augmentation.

**Fig. 3:**
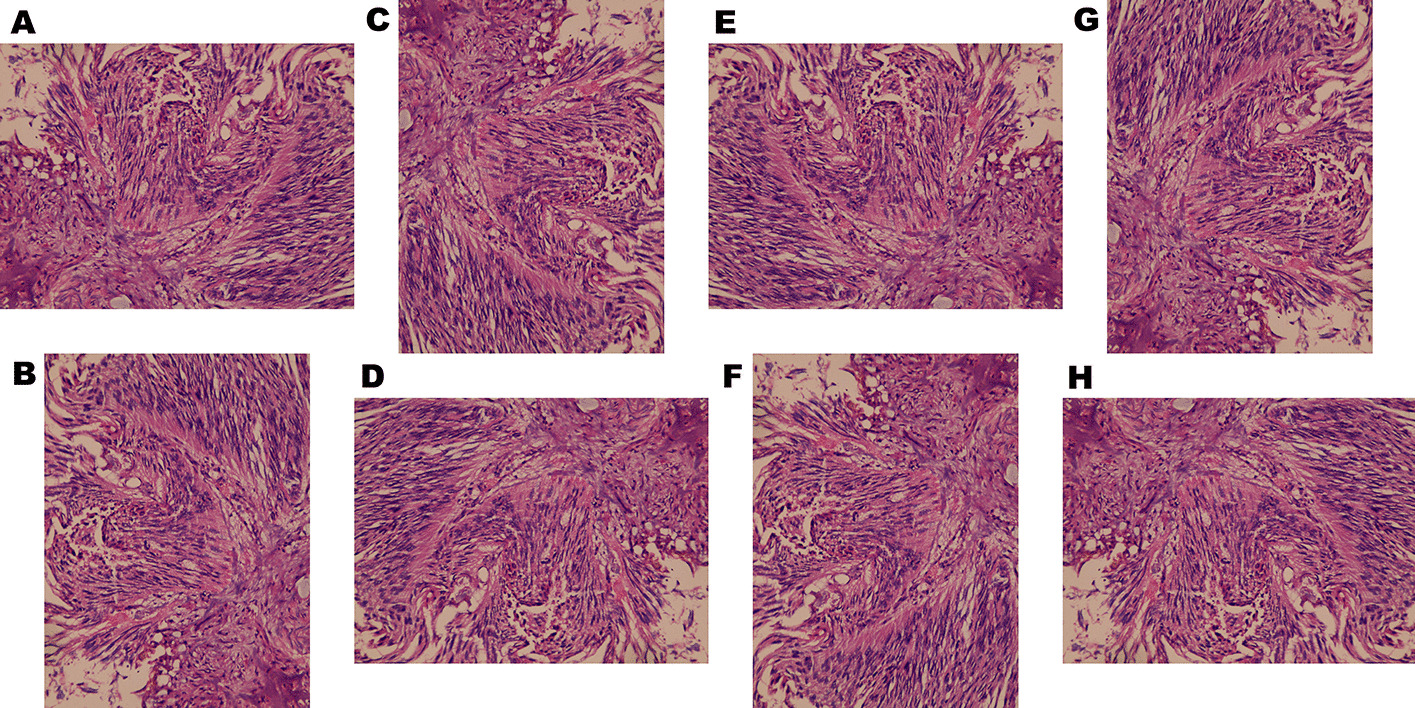
An example of image augmentation by rotation and flipping. An example of eight-fold augmentation by rotation and flipping; **A** Original urothelial cell carcinoma image; **B**–**D** Rotation of the original image by 90°, 180°, and 270° to produce three more images; **E**–**H** Flipping of the previous four images to produce four more images

We augmented the number of images eight folds by flipping and rotating the original images by 90°, 180°, and 270° (Fig. 3). Invariance to these geometric transformations is inherent to the practice of human pathologists. Since synthetic images may substantially differ from transformed images [23, 24], generative augmentation was not included in the present work.

**Fig. 4:**
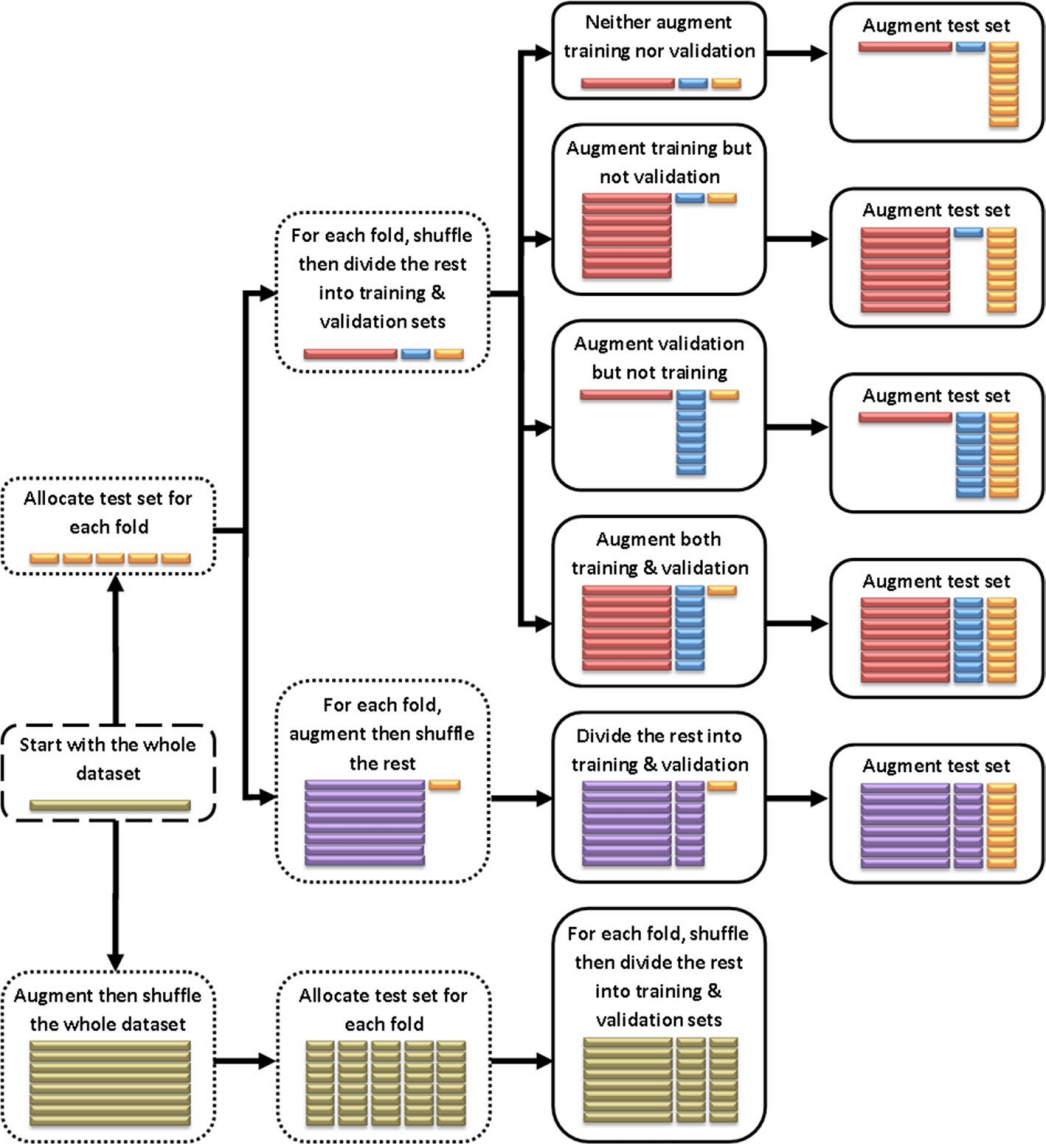
The 11 ways of data augmentation that were compared. The flowchart hierarchically illustrates the steps to implement the final 11 ways to apply data augmentation. Colored packets represent and are proportional to parts of the dataset. Red, blue, and orange packets represent independent training, validation, and testing data, respectively. Purple packets represent training and validation data when some training images are derived by augmenting some parent validation images and vice versa. Brown packets represent the three subsets when each subset contains some augmentation derivatives of some parent images in the other two subsets. Dashed-outline box = starting point; dotted-outline boxes = intermediate steps; solid-outline boxes = final 11 ways to apply data augmentation which were evaluated

We explored the effects of skipping augmentation; applying augmentation to different subsets of the whole dataset (the training set, the validation set, the test set, two of them, or all of them); and applying augmentation at different time points (before, during, or after dividing the dataset into three subsets). Different combinations of the above possibilities resulted in 11 ways to apply augmentation. Figure 4 is a flowchart that illustrates the steps to implement these ways. Augmentation before test set isolation was expected to leak information between the three subsets, leading to optimistic results [9]. However, we included it in the comparison for the sake of theoretical completeness.

The final preprocessing step was image resizing. Three image sizes were needed for the four CNNs in our work: 299 × 299 pixels for Inception-v3, 227 × 227 pixels for SqueezeNet, and 224 × 224 pixels for both ResNet-101 and GoogLeNet.

### Model building and testing

Dataset augmentation, resizing of images, and deep learning were implemented using MathWorks® MATLAB® on a Microsoft® Windows Dell™ Inspiron™ 15–7577 computer. The hardware comprised an Intel® Core™ i7-7700HQ processor, an 8-gigabyte memory, and an NVIDIA® GeForce® GTX 1050 Ti graphics card with a 4-gigabyte discrete memory.

We evaluated four of the CNNs that had been made available by the MathWorks Neural Network Toolbox Team [33]. These four CNNs were Inception-v3, ResNet-101, GoogLeNet, and SqueezeNet. Their architectures had been originally described in [34–37], respectively. All four CNNs had been pre-trained on subsets of the ImageNet dataset, which is a large dataset of annotated photographs of diverse objects [38, 39]. The specifications of these CNNs along with their performances (when evaluated on ImageNet images) are documented at [40].

**Fig. 5:**
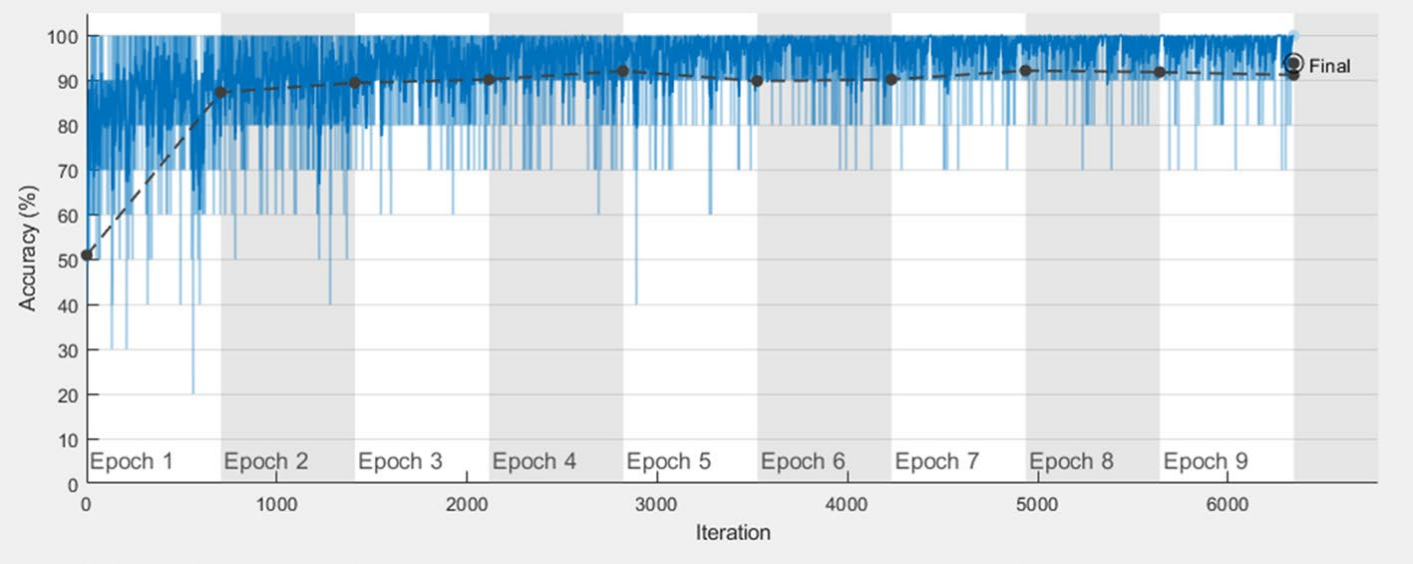
An example graph of the complete training progress to build one model. A graph generated by MathWorks® MATLAB® representing the progress during one fold of training. The black plot shows the validation accuracy while it rises from 50.89% (the baseline) up to 93.84% (at the end). The blue plot corresponds to the training accuracy. This fold took 195 min and 25 s to finish

Since the CNNs were pre-trained, the last three layers (fully connected layer, softmax layer, and classification layer) in each CNN were reset before the first training epoch. This enabled the CNN to be fine-tuned by the new dataset. During training, images are fed to the CNN in small groups called ‘mini-batches’. As the number of images in each mini-batch increases, training takes less time but requires more memory. We set the mini-batch size to 10 images due to the limited memory. We adopted the stochastic gradient descent algorithm with a momentum of 0.9 and a learning rate of 0.0001 as an ‘optimization algorithm’, which is the mechanism that tunes the CNN parameters to improve the model during training. L2 regularization, which is a technique that decreases overfitting, was applied with a factor of 0.0001. The validation set was used after each training epoch to gauge the model progress in terms of validation accuracy. Training stopped if the last five epochs did not improve the model or the total number of epochs reached 50. The training set was shuffled at the beginning of each epoch, so mini-batches differed between epochs. Figure 5 shows an example graph of the complete training progress to build one model.

The different ways of applying augmentation resulted in six models per CNN (each model was built five times for five-fold cross-validation). The training times, epoch counts, and validation accuracies were registered. Except when augmentation was applied to the whole dataset before test set allocation, all models were tested on both non-augmented and augmented test sets. Consequently, we ended up with 44 groups of testing results (× five folds) to analyze.

### Statistical analysis

Data to analyze were organized in a Microsoft® Excel® 2007 workbook. Statistical methods were implemented on StataCorp LP Stata®/IC 13.0 and MedCalc® 15.8 (MedCalc Software, Ostend, Belgium).

For the five folds of each model, the mean of the training-epoch counts and that of the training times were calculated. The 95% confidence interval of the mean was calculated by assuming a Student’s t-distribution with four degrees of freedom.

Model validation accuracy was estimated to help explain the results. Model testing performance was evaluated using four metrics: accuracy, sensitivity (recall), specificity, and area under the receiver operating characteristic curve (ROC AUC). Positive and negative predictive values and F_1_ score were skipped because they depend on prevalence. Although accuracy is also prevalence-dependent, the class sizes in our dataset are nearly equal, making accuracy an approximation of balanced accuracy (the mean of sensitivity and specificity) [41, 42].

A simplified explanation of these metrics in the context of our work is as follows. Accuracy is simply the rate by which the model correctly classifies images. Sensitivity is the rate by which the model correctly classifies UCC images as positive for malignancy, while specificity is the rate by which the model correctly classifies inflammation images as negative for malignancy. The clinical importance of each of sensitivity and specificity varies according to the situation in which the diagnostic test is applied. For example, sensitivity is vital for community-based screening tests, because minimizing missed cases (false negatives) is a priority for these tests. On the contrary, if the decision of a risky intervention depends on a diagnostic test, then this test should be highly specific to minimize undue interventions (false positives). Accuracy, sensitivity, and specificity are all dependent on the probability threshold used by the model for classification, which was 0.5 in our work. The receiver operating characteristic curve traces the trade-off between sensitivity and specificity as this probability threshold varies from 0 to 1. ROC AUC measures the discriminative ability of the model. It is mathematically equivalent to the probability by which the model will correctly discriminate between a randomly chosen UCC image and a randomly chosen inflammation image [42, 43].

Let TP_k_, TN_k_, FP_k_, and FN_k_ be the counts of true positive, true negative, false positive, and false negative results in the kth fold, respectively. Accuracy, sensitivity, and specificity were estimated using the following formulas:

$${\text{Accuracy = }}\frac{{\mathop \sum \nolimits_{{\text{k = 1}}}^{{5}} {\text{(TP}}_{{\text{k}}} {\text{ + TN}}_{{\text{k}}} {)}}}{{\mathop \sum \nolimits_{{\text{k = 1}}}^{{5}} \left( {{\text{TP}}_{{\text{k}}} {\text{ + TN}}_{{\text{k}}} {\text{ + FP}}_{{\text{k}}} {\text{ + FN}}_{{\text{k}}} } \right)}}$$

$${\text{Sensitivity (Recall) = }}\frac{{\mathop \sum \nolimits_{{\text{k = 1}}}^{{5}} {\text{TP}}_{{\text{k}}} }}{{\mathop \sum \nolimits_{{\text{k = 1}}}^{{5}} \left( {{\text{TP}}_{{\text{k}}} {\text{ + FN}}_{{\text{k}}} } \right)}}$$

$${\text{Specificity = }}\frac{{\mathop \sum \nolimits_{{\text{k = 1}}}^{{5}} {\text{TN}}_{{\text{k}}} }}{{\mathop \sum \nolimits_{{\text{k = 1}}}^{{5}} \left( {{\text{TN}}_{{\text{k}}} {\text{ + FP}}_{{\text{k}}} } \right)}}$$

For these formulas, binomial exact 95% confidence intervals were calculated by considering the counts in the denominators and numerators to be the counts of trials and successes, respectively. The prediction probabilities for all of the testing images of the five folds were pooled before being used in receiver operating characteristic curve analysis. ROC AUC and its binomial exact 95% confidence interval were calculated. This method for estimating ROC AUC penalizes models that have poor calibration across folds, as opposed to computing ROC AUC for each fold separately then taking the mean [44].

After exclusion of models where augmentation was applied before test-set allocation, and exclusion of metrics from non-augmented test sets, 20 groups of testing metrics were left. These were stratified by CNN, and then Pearson’s correlation was evaluated between each metric and the logarithmic transformation of the mean training time. The logarithmic transformation was employed since the performance metrics have upper limits, unlike the training time.

## Results

**Fig. 6:**
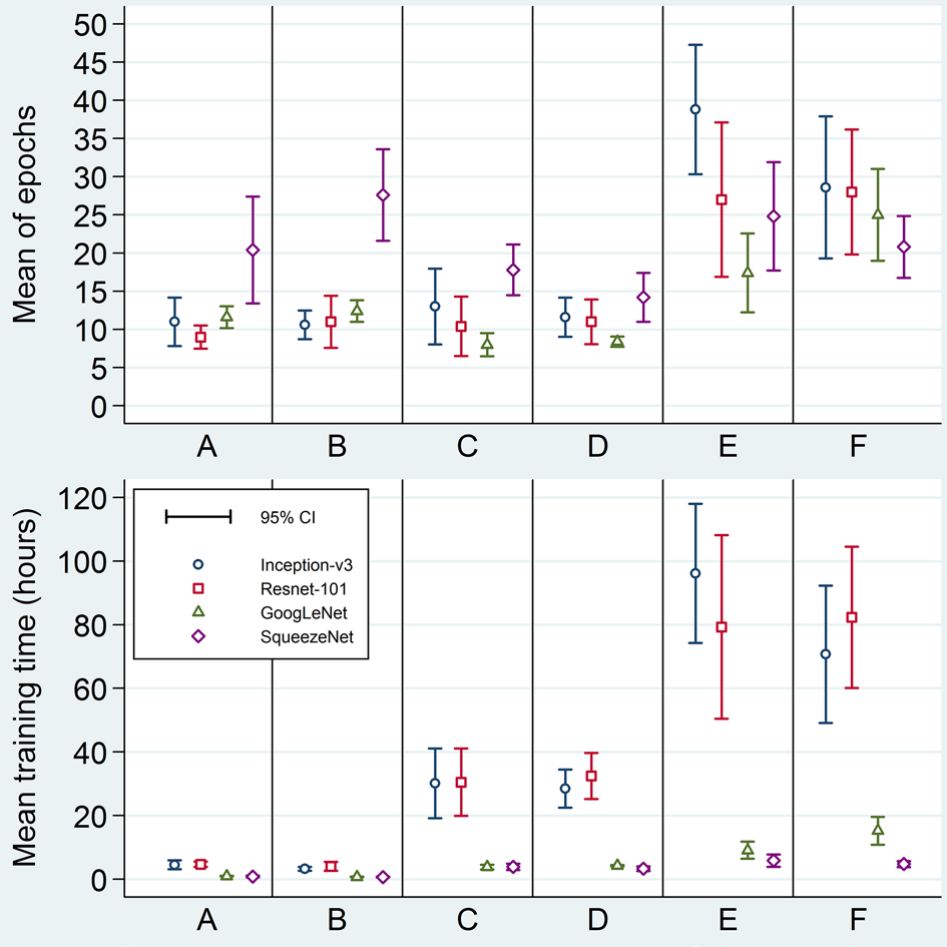
Mean training-epoch counts and mean training times. Mean training-epoch counts (above) and mean training times (below) for the 24 studied models. The four different symbol shapes/colors correspond to the four networks. Error bars are 95% confidence intervals. Horizontal axis labels stand for different ways of applying augmentation: A = Make three sets, then augment validation; B = Make three sets; C = Make three sets, then augment training; D = Make three sets, then augment both training and validation; E = Separate test set, augment the rest, then make two other sets; F = Augment all before making three sets. CI = confidence interval

The total count of training epochs per fold ranged 7—48; i.e., the maximum limit of 50 epochs was not reached. For each model, the mean epoch count per fold is shown in Fig. 6 and Additional file 1: Table S1. Mean training time ranged 0.72—96.11 h (Fig. 6; Additional file 1: Table S2). Shortest, intermediate, and longest times were consistently observed when the training set was not augmented (A and B in Fig. 6), augmented after separating the validation set (C and D in Fig. 6), and augmented before separating the validation set (E and F in Fig. 6), respectively. Inception-v3 and ResNet-101 took considerably more time than GoogLeNet and SqueezeNet.

**Fig. 7:**
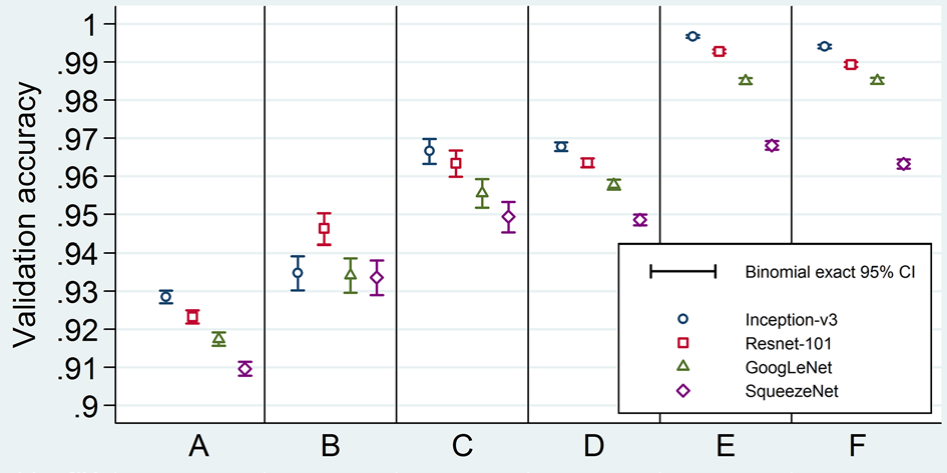
Model validation accuracies. Validation accuracies for the 24 studied models. The four different symbol shapes/colors correspond to the four networks. Error bars are binomial exact 95% confidence intervals. Horizontal axis labels stand for different ways of applying augmentation: A = Make three sets, then augment validation; B = Make three sets; C = Make three sets, then augment training; D = Make three sets, then augment both training and validation; E = Separate test set, augment the rest, then make two other sets; F = Augment all before making three sets. CI = confidence interval

**Fig. 8:**
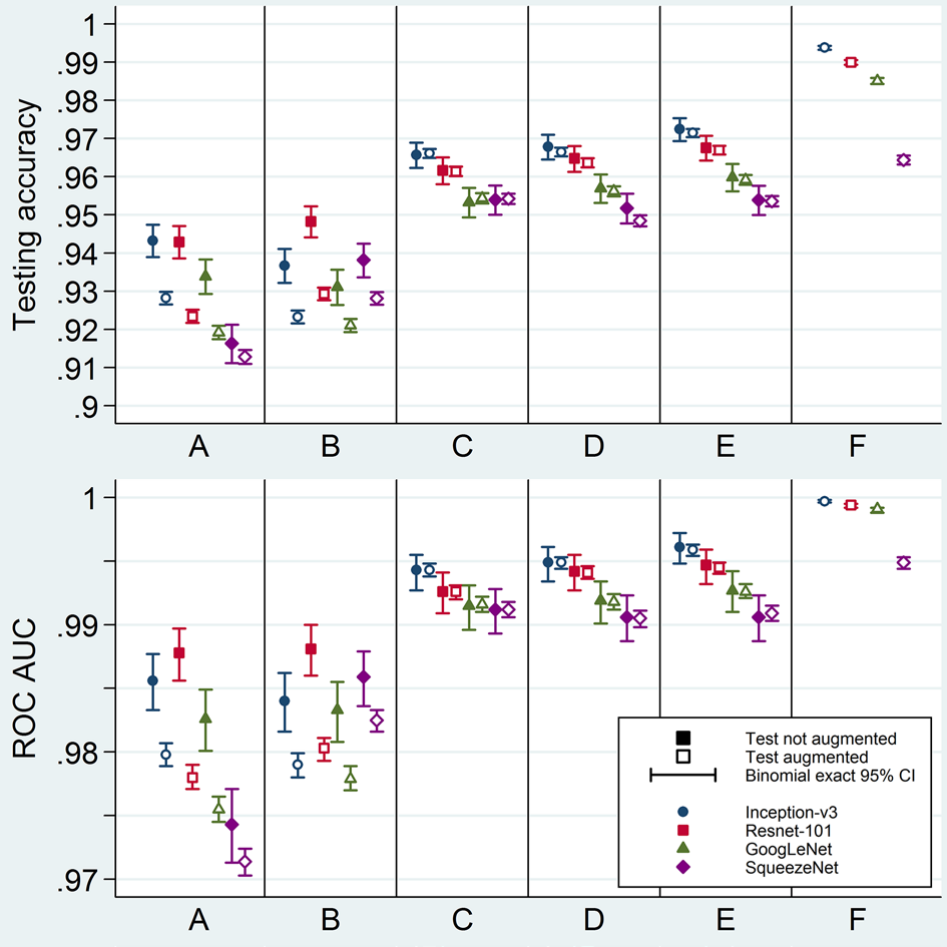
Model testing performance metrics: testing accuracy and area under receiver operating characteristic curve. Testing accuracy (above) and ROC AUC (below) for the 44 tests. The four different symbol shapes/colors correspond to the four networks. Solid and hollow symbols represent non-augmented and augmented test sets, respectively. Error bars are binomial exact 95% confidence intervals. Horizontal axes labels stand for different ways of applying augmentation: A = Make three sets, then augment validation; B = Make three sets; C = Make three sets, then augment training; D = Make three sets, then augment both training and validation; E = Separate test set, augment the rest, then make two other sets; F = Augment all before making three sets. CI = confidence interval; ROC AUC = area under the receiver operating characteristic curve

Validation accuracy results are shown in Fig. 7 and Additional file 1: Table S3. Augmenting the validation set alone lowered the validation accuracy (A in Fig. 7), while augmenting the training set by any way raised the validation accuracy (C–F in Fig. 7). However, this rise was more marked when augmentation was done before allocating the validation set. In other words, information leakage led to the highest validation accuracy (E and F in Fig. 7). Discrepancy between validation and testing accuracies was present only when augmentation was done between test-set and validation-set allocations. For these models, the validation accuracies were much higher than their testing counterparts (E in Figs. 7 and 8).

**Fig. 9:**
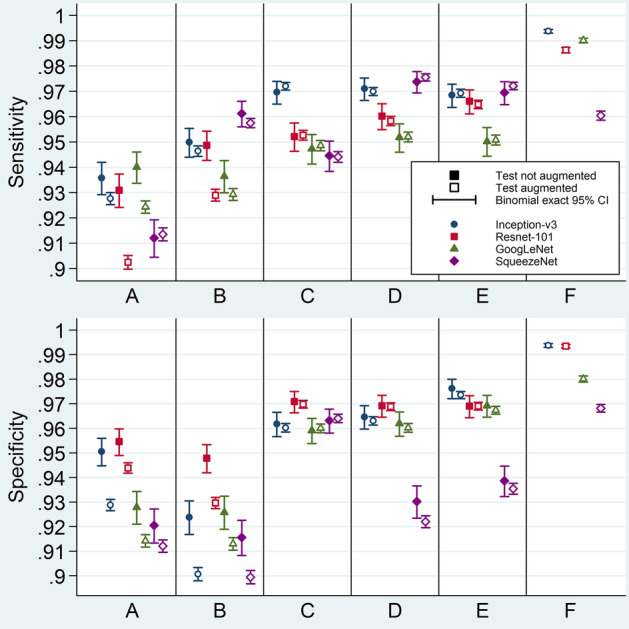
Model testing performance metrics: sensitivity and specificity. Sensitivity (above) and specificity (below) for the 44 tests. The four different symbol shapes/colors correspond to the four networks. Solid and hollow symbols represent non-augmented and augmented test sets, respectively. Error bars are binomial exact 95% confidence intervals. Horizontal axes labels stand for different ways of applying augmentation: A = Make three sets, then augment validation; B = Make three sets; C = Make three sets, then augment training; D = Make three sets, then augment both training and validation; E = Separate test set, augment the rest, then make two other sets; F = Augment all before making three sets. CI = confidence interval

**Fig. 10:**
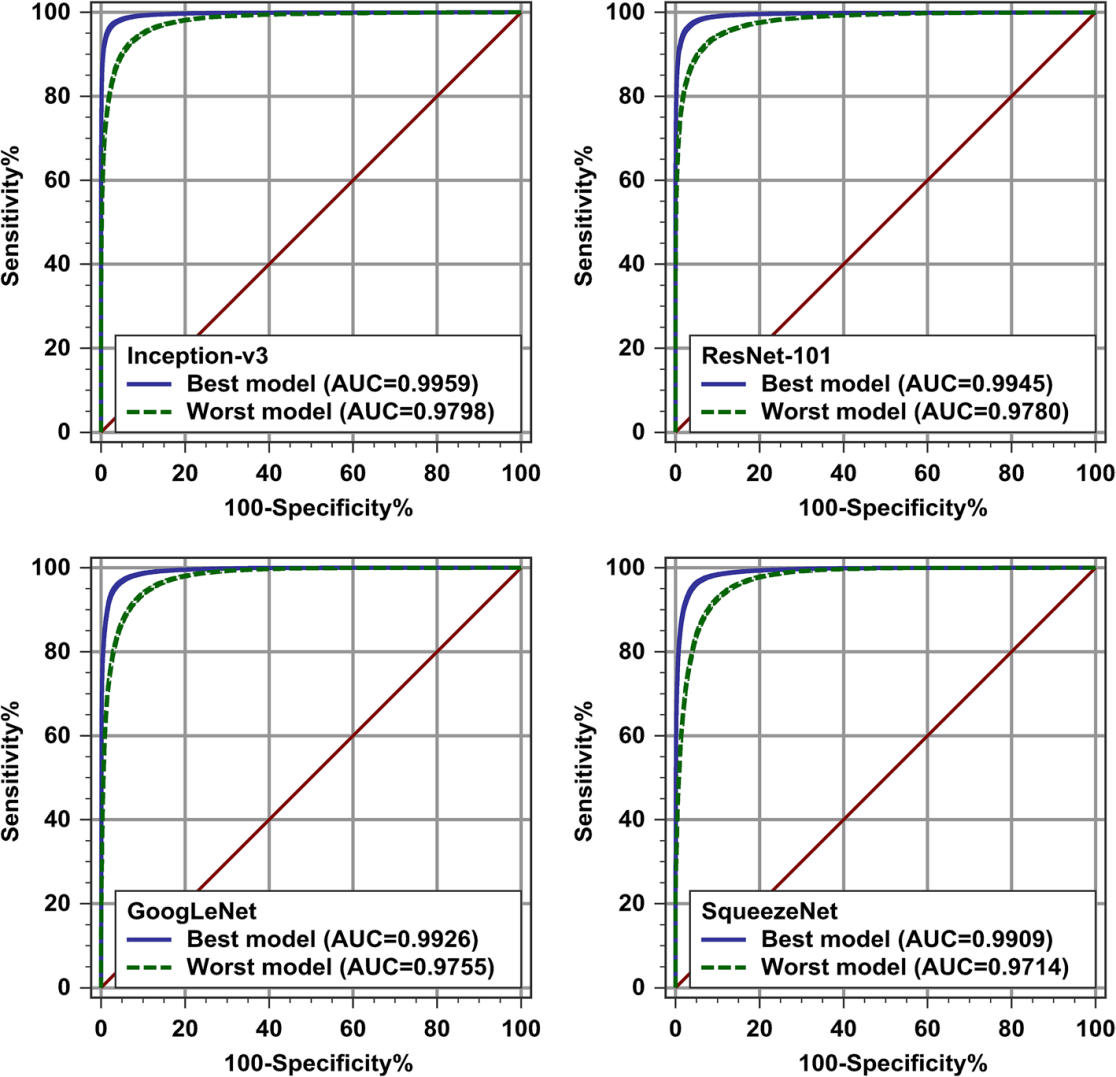
Best and worst receiver operating characteristic curves. Best (blue solid curves) and worst (green dashed curves) receiver operating characteristic curves for each of the four studied networks. Augment-first models and non-augmented test set results were excluded. AUC = area under the curve

Regarding model testing performance, ranges of accuracy, sensitivity, specificity, and ROC AUC were 91.28–99.38%, 90.25–99.38%, 89.95–99.38%, and 0.9714–0.9997, respectively (Figs. 8, 9, and 10; Additional file 1: Tables S4, S5, S6, and S7). After exclusion of the augment-first models and the non-augmented-test-set metrics, the upper limits of the previously-mentioned ranges decreased to 97.15%, 97.55%, 97.36%, and 0.9959, respectively.

For models tested on both non-augmented and augmented test sets, metric estimates were generally the same for both kinds of test sets except when the training set was not augmented. For the latter models, augmented-test-set metrics were remarkably lower (A and B in Figs. 8 and 9). Because augmented-test-set metrics are theoretically less biased, they will be the focus of the rest of the results section.

In general, the testing metrics were lowest when the training set was not augmented (A and B in Figs. 8 and 9) and highest for the augment-first models (F in Figs. 8 and 9). For the rest of the models, augmentation after test-set allocation and before separating the validation set yielded a slightly better testing performance (E in Figs. 8 and 9).

From the CNN point of view, Inception-v3 had the best overall testing performance, followed by ResNet-101, then GoogLeNet, and finally SqueezeNet (Fig. 8; Additional file 1: Tables S4 and S7). However, SqueezeNet had an exceptionally high sensitivity but at the cost of a low specificity. ResNet-101, on the contrary, excelled at specificity but with a low sensitivity (Fig. 9; Additional file 1: Tables S5 and S6).

**Fig. 11:**
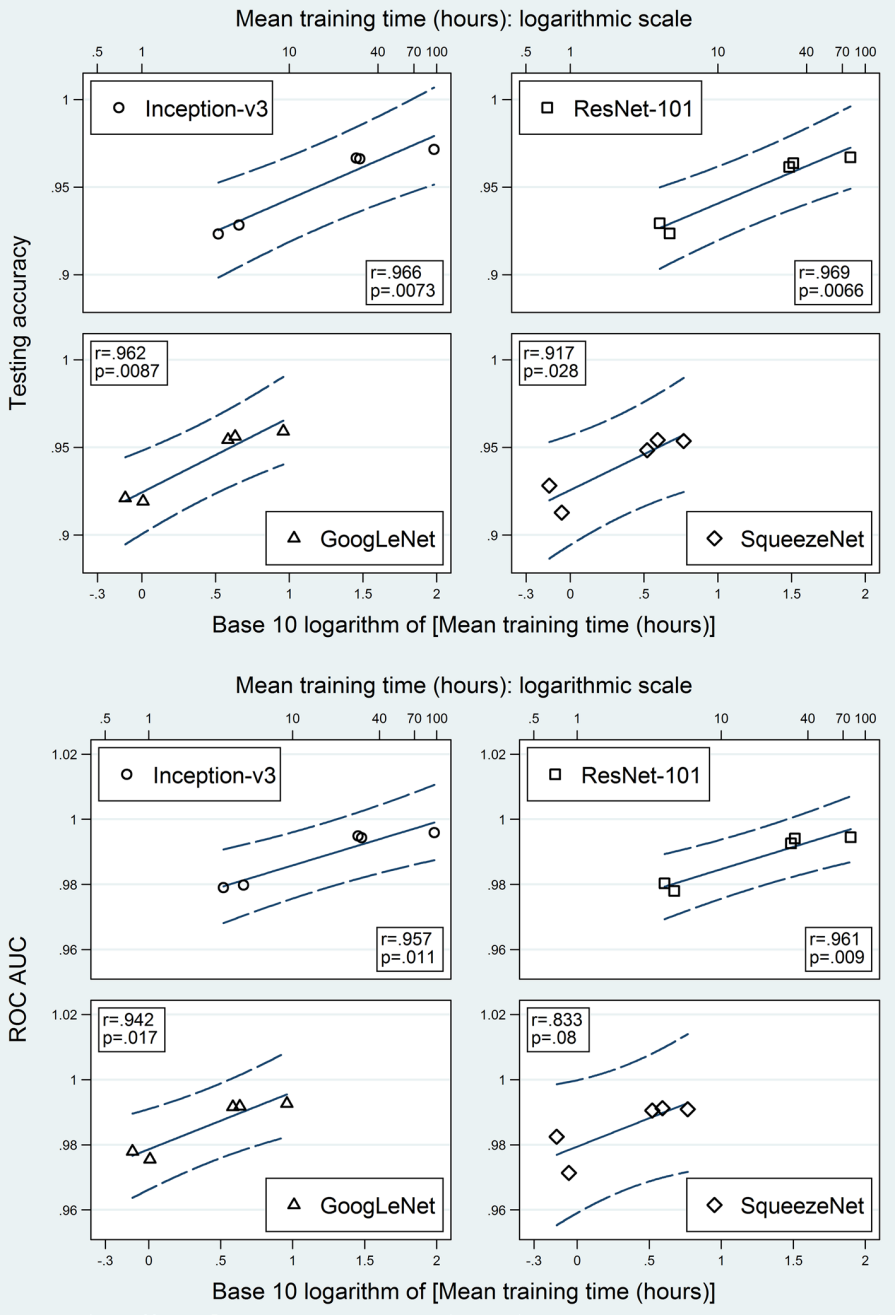
Testing accuracy and area under receiver operating characteristic curve versus mean training time. Scatter plots between testing accuracy (above) and ROC AUC (below) on one hand and the logarithmic transformation of the mean training time on the other hand, stratified by network. Augment-first models and non-augmented test set results were excluded. Blue solid lines are fitted regression lines. Blue dashed curves are the 95% confidence bands of the forecast (which include the prediction uncertainty of both the mean and the residual). Pearson’s product-moment correlation coefficient (r) and its p value are shown for each plot. ROC AUC = area under the receiver operating characteristic curve

**Fig. 12:**
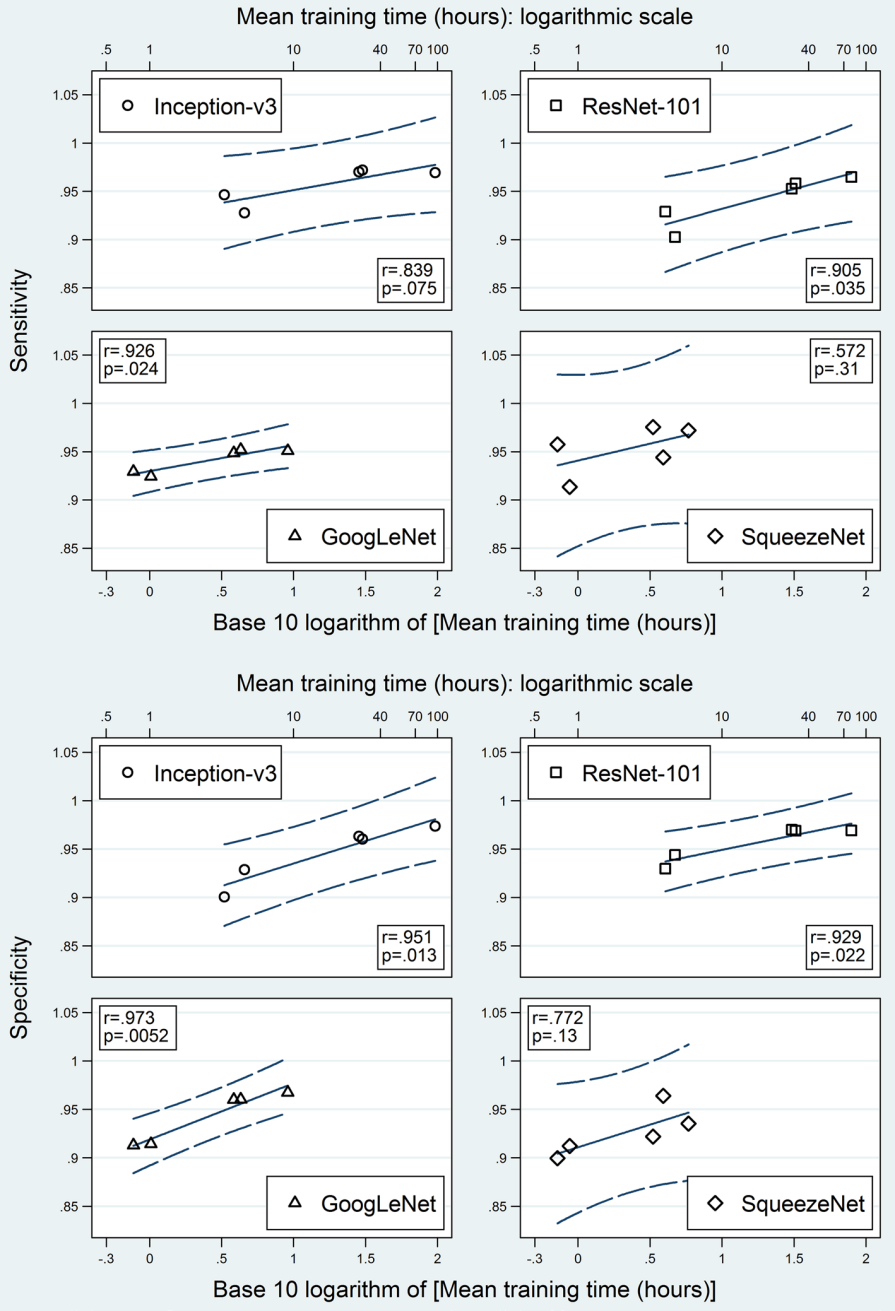
Sensitivity and specificity versus mean training time. Scatter plots between sensitivity (above) and specificity (below) on one hand and the logarithmic transformation of the mean training time on the other hand, stratified by network. Augment-first models and non-augmented test set results were excluded. Blue solid lines are fitted regression lines. Blue dashed curves are the 95% confidence bands of the forecast (which include the prediction uncertainty of both the mean and the residual). Pearson’s product-moment correlation coefficient (r) and its p value are shown for each plot

After further exclusion of the augment-first models, all four testing metrics revealed a strong correlation with the logarithm of the mean training time when stratified by CNN (Figs. 11 and 12). Ranges of Pearson’s correlation coefficients for accuracy, sensitivity, specificity, and ROC AUC were 0.917–0.969, 0.572–0.926, 0.772–0.973, and 0.833–0.961, respectively. SqueezeNet had the lowest coefficients for all four metrics. Except for the sensitivity of Inception-v3 and the sensitivity, specificity, and ROC AUC of SqueezeNet, all coefficients had p values < 0.05.

## Discussion

### Analysis of our results

#### Training-set augmentation

Substantially lower results were obtained when the training set was not augmented. This is not surprising, because training CNNs for histopathology image analysis typically needs a vast amount of labeled patches, much more than is available in our small dataset. Since the lack of adequate labeled patches is a standard problem in digital pathology, routine training-set augmentation is recommended.

Setting aside the optimistic augment-first method, the three remaining methods in which the training set was augmented yielded comparable results. Still, the best method was augmenting the training and the validation data together before validation-set allocation. Information leakage from the validation set to the training set enriched the latter, leading to a better model. Meanwhile, information leakage the other way round did occur, as indicated by the optimistic validation accuracy. However, the deflection (rather than the absolute value) of the validation accuracy is what stops the training. So, the ‘peeping’ validation set was still capable of preventing overfitting.

#### Test-set augmentation

An expected effect of augmenting the test set is narrower confidence intervals. But, apparently, test-set augmentation has another advantage in certain situations. When the training set was not augmented, the augmented-test-set metrics were lower than their non-augmented counterparts. This is corroborated by the observation that when the training set was not augmented, the validation accuracy also declined upon augmenting the validation set. Therefore, test-set augmentation is recommended for both a more realistic and a less uncertain estimation of the true generalization ability of the model.

It should be noted that another type of test-set augmentation, commonly known as ‘test-time augmentation’, can be used to actually boost the model performance. It is done by averaging the predictions for all transformations of an image [25]. This results in a single prediction for the original image along with its transformations. Thus, the total final count of predictions is the same as that of the data points in the non-augmented test set (see [45] below in the related work for an example). This resemblance between the two concepts may be confusing, but the deep-learning terminology is still evolving. The two concepts are not mutually exclusive; they can be used side by side to improve both model performance and evaluation, respectively.

#### Relationship between training time and performance

Holding the computational resources fixed, the improvement of the model performance may come at the expense of more training time. To assess the time-cost-effectiveness for the different examined ways of data augmentation, we plotted the performance metrics against the logarithmic transformation of the training time. For each CNN, a linear association was found between each performance metric and the logarithm of the training time. The slope of each fitted regression line may be directly used to express the time-cost-effectiveness of changing the augmented subset. We think that this type of plot may be used as well to quantify the time-cost-effectiveness of other categories of model-building manipulations (e.g. changing the resolution of patches or changing the method of transfer learning). However, some manipulation categories may have a more complex relationship with training time.

### Related work

In order to place our work in context, we reviewed the related literature from two orthogonal perspectives. First, we searched for answers to our research question in studies focusing on data augmentation (not necessarily bladder cancer histopathology). Second, we present an overview of studies that applied deep learning to histopathology images of bladder cancer, with a special attention to whether/how data augmentation was used.

#### Studies comparing the outcome of data augmentation when applied to the various subsets of the dataset

The vast majority of papers focusing on data augmentation are concerned with exploring and comparing augmentation techniques [25] rather than to which dataset partition(s) augmentation should be applied. Moreover, in many papers the validation set did not control the number of training epochs, or was absent altogether. In many others, the authors did not describe exactly which data were augmented. Still in others, synthetic data were generated for augmentation. We were left with a few studies that could be compared to ours, but none of which systematically compared the augmentation of all possible subsets as we did. In general, none of the comparable studies contradicted with our results.

##### No augmentation versus training-set augmentation

Laves et al. [46] segmented laryngeal endoscopic images into seven classes using four different CNNs: U-Net, SegNet, ENet, and ERFNet. The dataset contained 200, 100, and 100 images for training, validation, and testing, respectively. Data augmentation by horizontal flipping and rotation (within ± 10°) increased the training set ten-fold. As a result, the mean Jaccard index was improved for all four CNNs, from 73.6%, 67.3%, 72.4%, and 73.5% to 76.8%, 72.3%, 78.4%, and 81.6%, respectively.

Jin et al. [47] classified lymph node histopathology images according to the presence or absence of metastatic breast cancer tissue. The training, validation, and test sets contained 262,144, 32,768, and 32,768 images, respectively. While they augmented the training set by shifting, rotation, and flipping, they did not mention the augmented training set size. Augmentation improved accuracy from 76.4% to 78.8%, and ROC AUC from 0.854 to 0.884. The improvement is slight, may be due to the large pre-augmentation dataset size.

Hao et al. [48] detected prostate cancer in diffusion-weighted magnetic resonance imaging slices. The dataset was composed of 5300 training, 2500 validation, and 2328 testing slices. Through augmenting only the training set two-fold (10,600 slices), they systematically compared many hyperparameter settings for each of five augmentation techniques: random rotation, horizontal flipping, vertical flipping, random cropping, and translation. Both a shallow and a deep CNN were used. Interestingly, the shallow CNN generally outperformed the deep one. Moreover, augmentation did not consistently improve the ROC AUC. Instead, this depended on the augmentation technique, the CNN, and even the augmentation hyperparameter setting. Generally, the best ROC AUC improvements were obtained by random rotation and translation for the shallow and deep CNNs, respectively. This highlights the importance of tailoring the augmentation technique according to both the classification problem and the CNN architecture.

##### No augmentation versus test-set augmentation

When we applied augmentation to the test set, performance metrics were averaged with the aim of decreasing the uncertainty rather than improving the model performance. As mentioned above, ‘test-time augmentation’ is a different, yet closely related, concept capable of improving the model [25]. For example, Hoar et al. [45] segmented prostate cancer in multiparametric magnetic resonance imaging slices using a modified-VGG CNN. They augmented the test set 16-fold by scaling, rotation, and horizontal flipping. For each pixel in each test image, the multiple generated predictions were averaged. Test-time augmentation improved the Dice similarity coefficient from 54 to 56% without changing the standard error (6%).

##### No augmentation versus training-set and validation-set augmentation

Li et al. [49] detected notable peripheral retinal lesions in fundus images using four CNNs: Inception-ResNet-v2, Inception-v3, ResNet-50, and VGG-16. The training, validation, and test sets contained 3504, 751, and 750 images, respectively. The training and validation sets were augmented to 17,500 and 3750 images, respectively, by brightness shifting, rotation, and flipping. Augmentation improved the sensitivity, specificity, accuracy, and ROC AUC for all four CNNs.

Zhou et al. [50] used Inception-v3 to detect diatoms. Training and validation sets contained 2634 and 618 tiles from 43 and 10 slides, respectively, while 5 slides were kept aside for testing. The training and validation sets were augmented to 13,170 and 3090 tiles, respectively, by rotation and changing contrast and brightness. When the performance was evaluated on the validation set, augmentation improved both the accuracy and the ROC AUC, regardless of whether the CNN was trained from scratch or pre-trained on ImageNet. Unfortunately, no testing metrics were reported.

##### Whole-dataset augmentation before splitting into three partitions

It is well-known that information leakage from training to testing results in over-estimated performance metrics [9]. However, we encountered a paper that deliberately augmented the dataset before partitioning. Zeng and Zhang [51] detected breast cancer in histopathology images using Google Cloud AutoML Vision. The original dataset consisted of 198,738 negative and 78,786 positive patches from 279 patients. To balance the dataset, 60,000 positive patches from 227 patients were augmented three-fold by rotation. Thirty other patients were set aside to provide 34,128 non-augmented patches (independent test set). The remaining 363,396 patches (which included those augmented) were split into training, validation, and test sets in a ratio of 8:1:1. For the ‘peeping’ test set, the F_1_-score and the balanced accuracy were 86.4% and 85.3%, respectively. For the independent test set, these figures decreased to 77.1% and 84.6%, respectively. The F_1_-score decline was more pronounced, probably because the augmentation was confined to only the positive class.

#### Studies pertaining to bladder cancer histopathology

A systematic search was done in both the PubMed [52] and the IEEE [53] databases. References cited in relevant studies were also scrutinized. The papers found, although few, can be considered a representative sample for studies that apply deep learning to histopathology in general. They clearly demonstrate that data augmentation is underused, inconsistently implemented, and ambiguously reported. Meanwhile, they report performance levels that are still too modest to import to the clinic. The method and performance of deep learning in each of these studies are overviewed here, categorized by aim. Table 1 summarizes the dataset criteria in each study.

**Table 1:** Criteria of datasets used in studies applying deep learning to bladder cancer histopathology images

Reference	Dataset source(s)	Count and pathology of patients/slides	Stain	Magnification	Count of images (tiles) (patches)	Dimensions and selection of images (tiles) (patches)	Data augmentation method(s)	Training (: validation) : testing ratio
[29]^a^	TCGA	≈ 500 slides of UCC or adjacent normal cuts	H&E	20 ×	4711 normal and 73,425 cancer (depending on slide-level labels)	512 × 512 Non-overlapping After background removal	None	70:30 (of slides) Stratified
[29]^b^	TCGA	388 UCC slides	H&E	Not mentioned	185,064 total	512 × 512 Non-overlapping Excluding normal tiles	None	70:30 (of slides)
[54]	Not mentioned	Eight bladder biopsy slides Pathology was not mentioned	H&E	40 ×	Not mentioned	For training and validation: 64 × 64 at 10 × Non-overlapping After background removal For testing: 64 × 64 by a sliding window with 8-pixel steps	None	Not mentioned
[55]	The Ohio State University	39 T1 bladder cancer^c^ slides	H&E	40 ×	Excluding background tiles: 13,606 training, 1360 validation, and 1359 testing	512 × 512 Non-overlapping Including background	None	31:4:4 (of slides) Non-stratified for tiles/classes
[56]	University Hospital of Stavanger, Norway	32 UCC patients/slides	HES	400 × (100 × and 25 × by down-sampling)	139,861 (after augmentation) at each magnification level	128 × 128 400 × tiles: non-overlapping for all classes (including background) except muscle and stroma where 50% overlap was present 100 × and 25 × tiles: centered at corresponding 400 × tiles	For muscle and stroma training tiles only: rotation and flipping	Five-fold cross-validation (of patients) using only training and testing sets (no validation set)
[57]^d^	Three centers in the Netherlands	328 non-muscle invasive UCC specimens from 232 patients	H&E	20 ×	≈ 500,000 total	572 × 572 25% overlap Excluding patches with ≥ 75% background pixels	Random color variation, flipping, and mirroring of the training patches	60:20:20 (of patients)
[57]^e^	Three centers in the Netherlands	328 non-muscle invasive UCC specimens from 232 patients	H&E	20 ×	123,132 undefined, 564,710 low grade, and 493,374 high grade	224 × 224 25% overlap From regions of urothelium segmented by U-Net	Random flipping and mirroring of the training patches	60:20:20 (of patients)
[14]	TCGA and University of Florida Health Shands Hospital in the United States	913 UCC slides	H&E	40 ×	Training: 148,671 Validation: 8371 Testing: not mentioned	1024 × 1024 Randomly From manually partially annotated tumor and non-tumor regions Each has a binary annotation mask	Rotation, horizontal and vertical flips, and random crop Not mentioned to which data it was applied	620:193:100 (of slides)
[58]	Edinburgh hospitals	100 muscle-invasive UCC patients/slides	IF (PanCK, Hoechst)	20 ×	Not mentioned	Not mentioned	None	Not mentioned
[59]^f^	TCGA	100 UCC patients/slides	H&E	20 ×	Excluding testing: 79,747 tumor and 92,797 non-tumor	512 × 512 Non-overlapping Including background	Random rotation, zooming, flipping, and color-based During training	48:12:40 (of slides)
[59]^g^	TCGA	253 UCC patients/slides (124 low and 129 high tumor mutational burden)	H&E	For AP clustering: 2.5 × For feature extraction: 20 ×	125,358 total tumor tiles, from which AP clustering selected 11,164 representative tiles	For AP clustering: 128 × 128 Non-overlapping From segmented tumor For feature extraction: 1024 × 1024 Selected by AP clustering	None	Leave-one-out cross validation
[60]	University of Rochester Medical Center	1177 UCC images (460 stage Ta and 717 stage T1) Not mentioned if each image came from a separate slide	H&E	100 ×	Not mentioned	700 × 700 One to four images were cropped from the central part of each raw image	None	70:30 (after sampling 460 Ta and 460 T1 images^h^)
[61]	TCGA and local institution of the authors	Muscle-invasive UCC TCGA: 318 slides from 294 patients Local institution: 38 slides from 13 patients	H&E	10 ×	Training patches: 18,552, 68,880, 264,550, and 1,044,158 at effective 2.5 × , 5 × , 10 × , and 20 × , respectively Rest of patches: Not mentioned	300 × 300 (at effective 2.5 × , 5 × , 10 × , and 20 ×) Non-overlapping From manually annotated tumor regions	Random rotation, flipping, warping, brightness, and contrast During training	TCGA: 146:73:75 (of patients) Local institution: all testing
[15]	TCGA and University Clinic Hospital Erlangen	Muscle-invasive bladder cancer^i^ TCGA: 363 (training and validation) patients/slides Erlangen: 16 (testing) patients/slides	H&E	TCGA: Not mentioned Erlangen: 40 ×	TCGA: 807,943 total, but only a random 250,833 were used Erlangen: Not mentioned	512 × 512^j^ Non-overlapping From manually annotated tumor regions	Random flipping, mirroring, contrast / saturation / brightness changes, and cutouts Not mentioned to which data it was applied	TCGA: 90:10 (of slides) stratified
[62]^k^	The Stanford tissue microarray database	2139 bladder cancer^g^ slides (542 GATA3, 514 CK14, 544 S100P, and 539 S0084)	IHC	Not mentioned	Not mentioned	224 × 224 (Inception-v1) and 229 × 229 (Inception-v3, and Inception-ResNet-v2) Not mentioned how tiles were derived from slides	None	70:15:15 (of slides)
[62]^l^	The Stanford tissue microarray database	2137 bladder cancer^g^ slides (680 Score 0, 235 Score 1, 284 Score 2, and 938 Score 3)	IHC	Not mentioned	Not mentioned	224 × 224 (Inception-v1) and 229 × 229 (Inception-v3, and Inception-ResNet-v2) Not mentioned how tiles were derived from slides	None	70:15:15 (of slides)
[63]	TCGA	332 UCC patients Slide count was not mentioned	H&E	20 ×	Not mentioned	512 × 512 Non-overlapping From manually annotated tumor regions	Random horizontal and vertical flipping During training	Stratified three-fold cross-validation (of patients)
[64]	TCGA	381 UCC slides	H&E	For the lymphocyte CNN: 20 × For the necrosis CNN: 6.67 ×	Not mentioned	Non-overlapping For the lymphocyte CNN: 100 × 100 Excluding background For the necrosis CNN: 333 × 333	Only for the lymphocyte CNN: Random cropping^m^, color perturbing, rotation, and mirroring For training and testing separately	Not mentioned
[65]	TCGA	290 UCC patients/slides	H&E	20 ×	10,000 patches per slide	100 × 100 Non-overlapping	None	Not mentioned
[66]	Amsterdam University Medical Center	Non-muscle invasive UCC 359 and 281 patients for 1- and 5-year survival, respectively Slide count was not mentioned	H&E	20 ×	1-year: ≈ 5,500,000 (recurrence in 35%) 5-year: ≈ 4,400,000 (recurrence in 64%)	224 × 224 Non-overlapping From urothelium segmented by U-Net [57]	None	60:20:20 (of patients)

^a^Dataset to distinguish cancer from normal. Approximate figures were retrieved from graphs as they were neither mentioned accurately in the paper nor in the supplementary materials

^b^Dataset to classify *TP53* mutation status

^c^No specific histology was stated

^d^Dataset to segment urothelium

^e^Dataset to grade the segmented urothelium

^f^Dataset for tumor segmentation

^g^Dataset for patient-level tumor mutational burden classification into low or high categories

^h^Not stated if these were raw images or tiles

^i^UCC from TCGA, but histology not specified for the Erlangen cohort

^j^A supplementary figure suggests that tile resolution is 1 μm/pixel, i.e., 10 ×

^k^Dataset for biomarker classification

^l^Dataset for biomarker staining score classification

^m^Input patches were randomly cropped from a larger image. However, it is not clear how this does not contradict with subdividing the whole slide image into non-overlapping patches

*AP* Affinity propagation, *CNN* Convolutional neural network, *H&E* Hematoxylin and eosin, *HES* Hematoxylin eosin saffron, *IF* Immunofluorescence, *IHC* Immunohistochemistry, *PanCK* Pan-cytokeratin, *TCGA* The Cancer Genome Atlas, *UCC* Urothelial cell carcinoma

##### Cancer versus non-cancer image classification

Noorbakhsh et al. [29] used an Inception-v3-based CNN (pre-trained on ImageNet) for this (rather simple) task. While tile-level sensitivity and accuracy were about 95%, specificity was only about 75%. This somewhat poor performance may be due to the tiles being labeled according to their parent slide labels. No data augmentation was utilized.

##### Segmentation

Niazi et al. [54] reported results for differentiating between lamina propria, red blood cells, and inflammation on one hand and muscularis propria on the other hand. AlexNet, Inception-v3 (both pre-trained on the ILSVRC-2012 dataset), and stacked autoencoders achieved accuracies of 88%, 97%, and 80%, respectively. No data augmentation was utilized. In another work [55], the same group attempted eight-class segmentation using a modified U-Net. Pixel-level accuracies for classifying background, lamina propria, muscularis propria, mucosa, and red blood cells ranged 88–99%. Accuracies for classifying cautery and inflammation, on the other hand, ranged only 28–52%. Although muscularis mucosa was among the eight classes, too few tiles were available to determine its segmentation accuracy. While the authors did not use data augmentation, they attributed the poor performance in segmenting cautery and inflammation to the limited number of training tiles for these two classes.

Wetteland et al. [56] also attempted to segment six classes: urothelium, damaged tissue, stroma, blood, muscle, and background. Their model, based on the ImageNet-pre-trained VGG-16, achieved the best F_1_-score (96.5%) when designed to learn from three (rather than one or two) magnification levels for each training tile. While the higher-magnification tiles contain more small-detail information, the lower-magnification tiles contain more contextual information. Therefore this multiscale approach should not be considered as data augmentation. Indeed, data augmentation was implemented, but only for muscle and stroma training tiles.

Jansen et al. [57] used U-net to segment the urothelium. More urothelium was detected than the ground-truth, with false positive regions in 13% of the test samples. Data augmentation was used only for the training patches. Zhang et al. [14] also used U-net, but to generate tumor probability maps. While they reported using data augmentation, they did not specify to which dataset partition(s) it was applied. At a probability threshold of 0.3, pixel-level sensitivity and specificity were 94.8% and 95.3%, respectively.

As an initial step before quantifying tumor buds, Brieu et al. [58] applied a combination of CNN and random forest models to segment tumor cell clusters and detect the nuclei within. Without data augmentation, they achieved a Dice similarity coefficient of 86% for tumor segmentation and a Pearson’s r of 0.993 for nuclei detection.

Xu et al. [59] used a custom-designed light-weight CNN for tumor segmentation, and achieved a sensitivity of 90.65% and a Dice similarity coefficient of 90.76%. Data augmentation was used for the training set, but it is unclear if the validation set was also augmented.

##### Grading

Jansen et al. [57] used the ImageNet-pre-trained VGG-16 for grading UCC according to the WHO’04 system. Augmenting only the training data, 71% and 76% of the high-grade and the low-grade cases, respectively, were correctly graded. However, the inter-rater agreement between the model and the consensus of the three pathologists that defined the ground-truth (κ = 0.48) was comparable to the pairwise agreements between these pathologists (κ = 0.35; κ = 0.38; κ = 0.52).

##### Staging

Yin et al. [60] attempted differentiation between Ta and T1 UCC images by VGG-16 and VGG-19, both pre-trained on general images. No augmentation was used. Accuracies of 84% and 81%, and ROC AUCs of 0.926 and 0.912, were achieved by VGG-16 and VGG-19, respectively.

Harmon et al. [61] used ResNet-101 to predict lymph node metastasis. Data augmentation was used during training, but it is not obvious if this included the validation patches (which were obtained from different patients). Also, it is not obvious whether the number of training epochs was predefined or controlled by the validation performance. Four models were built, each using a different magnification level. Patch-level accuracies of 64.4%, 62.7%, 61.9%, and 58.8% were achieved by the 2.5 × , 5 × , 10 × , and 20 × models, respectively.

##### Molecular subtyping

Woerl et al. [15] trained a ResNet-50-based mibCNN to differentiate between four molecular subtypes. While they used augmentation, they did not specify if it was applied to the training set alone or also to validation and/or test sets (which were obtained from different slides). Although they had a total of > 800,000 tiles (before augmentation) available for training and validation, they reported that using more than ≈20,000 tiles did not improve the validation accuracy. They indeed used only ≈250,000 tiles and achieved slide-level validation and testing accuracies of 70% and 75%, respectively. It should be noted that the authors apparently used the ‘validation’ set for testing the model rather than for controlling the number of training epochs.

Khosravi et al. [62] classified non-augmented immunohistochemistry-stained images using pre-trained Inception-v1, Inception-v3, and Inception-ResNet-v2. The three CNNs achieved accuracies of 99%, 98%, and 85.5% at classifying four biomarker types; and 77%, 76%, and 58% at classifying four biomarker staining scores, respectively. The authors attributed the poor performance in the latter task to the subjectivity inherent to labeling staining scores by a human pathologist.

##### Genetic mutations

Noorbakhsh et al. [29] used Inception-v3 without data augmentation to detect *TP53* mutation. Tile-level and slide-level ROC AUC were 0.68 and 0.71, respectively. Loeffler et al. [63] used a modified ShuffleNet to detect patient-level single-gene mutations and signaling-pathway alterations. They reported augmenting the training data, but did not mention the presence of a validation set in the first place. Among 50 genes, *FGFR3*, *CTCF*, *ERBB2*, *CTCF*, *TP53*, and *ERBB2* achieved the highest ROC AUC, accuracy, sensitivity, specificity, F_1_-score, and Matthews’ correlation coefficient of 0.780, 98.2%, 76.7%, 99.7%, 61.8%, and 0.323, respectively. Among 12 pathways, *Wnt* achieved the highest ROC AUC of 0.68.

Xu et al. [59] classified UCC slides according to tumor mutational burden (high versus low). Their method starts by using ImageNet-pre-trained Xception to extract features, which are reduced by principle component analysis, and finally used to train a support vector machine. Instead of augmenting data, they used affinity propagation clustering to select only the representative tiles for training, reducing them from ≈125,000 to ≈11,000 tiles. This greatly shortened computational time, albeit for a slight performance loss (ROC AUC of 0.769 and 0.752, respectively).

##### Tumor-infiltrating lymphocytes

Saltz et al. [64] generated tumor-infiltrating-lymphocyte maps from whole-slide images by detecting lymphocyte-infiltrated patches using a semi-supervised CNN. Additionally, to avoid misclassifying the nuclei in the necrotic regions as lymphocytes, DeconvNet was used for necrosis segmentation. As for the former CNN, while the authors reported augmenting both the training and the testing data separately, they did not make a clear statement about augmenting the validation data. Regarding the latter CNN, no data augmentation was mentioned. Velmahos et al. [65] used the same tumor-infiltrating-lymphocyte-detection CNN but without an accompanying necrosis-segmentation CNN and without data augmentation. Unfortunately, no quantitative assessment results were reported for the performance of the CNNs used in these two studies.

##### Prediction of recurrence

Lucas et al. [66] attempted prediction of 1-year and 5-year recurrence-free survival using a two-step method. First, ImageNet-pre-trained VGG-16 was used for feature extraction without data augmentation. Next, the extracted features were fed to a bidirectional gated recurrent unit for classification. Accuracy, sensitivity, specificity, and ROC AUC were 61%, 50%, 65%, and 0.56 for the 1-year recurrence; and 67%, 93%, 38%, and 0.72 for the 5-year recurrence, respectively.

### Limitations

The principle limitation in our study is its simulative (as opposed to analytical) nature. This limitation greatly restricts the extrapolation of our conclusions. Only one trivial classification task for urinary bladder histopathology images was used as a benchmark for our research question. Data augmentation was done only by rotation and flipping. Only four pre-trained CNNs were picked as prototype examples. Training was done using a fixed set of hyperparameters. Indeed, the variables are countless, and manipulating any of them may provide different results.

## Conclusions

In the field of digital histopathology, we recommend data augmentation routinely to combat the deficiency in annotated datasets. Augmentation should include both the test set (after its allocation), and the remaining combined training/validation set (before being split into separate training and validation sets). While the latter maximizes the actual model performance, the former enables a less optimistic evaluation of this performance. Future research should try to generalize our results using other augmentation techniques (such as color transformations) and other deep-learning tasks as alternative benchmarks.

## Supplementary Information

**Additional file 1.** Supplementary Tables S1–S7 in portable document format (PDF). These tables replicate the results shown graphically in Figures 6–9, but as precise numbers.

**Additional file 2.** A Microsoft® Excel® workbook that contains an overview of the raw data for all of the 44 experiments.

**Additional file 3.** A Microsoft® Excel® workbook that details the raw data for the 20 experiments in which no test-set augmentation was done, including all of the image-classification output probabilities.

**Additional file 4.** A Microsoft® Excel® workbook that details the raw data for the 8 experiments in which either the test set was augmented alone (after its allocation) or augmentation of the whole dataset was done before test-set allocation. All of the image-classification output probabilities are included.

**Additional file 5.** A Microsoft® Excel® workbook that details the raw data for the 8 experiments in which both the training set and the test set were augmented after their allocation. All of the image-classification output probabilities are included.

**Additional file 6.** A Microsoft® Excel® workbook that details the raw data for the 8 experiments in which augmentation of the test-set was done after its allocation and the validation set was either augmented with the training set before their allocation or augmented without the training set after their allocation. All of the image-classification output probabilities are included.

## Data Availability

The histopathology image dataset that was used for this study is available in the Dryad repository at 10.5061/dryad.0cfxpnw5q [30]. The raw data for all of the experiments, including all of the image-classification output probabilities, are available as five supplementary Microsoft® Excel® 2007 workbooks at the website of the journal (Additional file 2, 3, 4, 5, and 6).

## Abbreviations

CNN: Convolutional neural network
ROC AUC: Area under the receiver operating characteristic curve
UCC: Urothelial cell carcinoma
