# Supplementary material for: Which data subset should be augmented for deep learning? a simulation study using urothelial cell carcinoma histopathology images
Source: BMC Bioinformatics. 2023 Mar 3;24:75. doi: 10.1186/s12859-023-05199-y (PMC9983182; doi:10.1186/s12859-023-05199-y)
Supplement: Supplementary file 1 — Additional file 1. Supplementary Tables S1–S7 in portable document format (PDF). These tables replicate the results shown graphically in Figures 6–9, but as precise numbers. [file 12859_2023_5199_MOESM1_ESM.pdf]

**File legend**

The present supplementary file contains Supplementary Tables S1—S7 in portable document format (PDF). These tables replicate the results shown graphically in Figures 6—9, but as precise numbers.

**Supplementary Table S1. Mean count of training epochs per fold with 95% confidence interval in parentheses**

| | Make 3 sets, then augment validation | Make 3 sets | Make 3 sets, then augment training | Make 3 sets, then augment both training and validation | Separate test set, augment the rest, then make 2 other sets | Augment all before making 3 sets |
|---|---|---|---|---|---|---|
| **Inception-v3** | 11(7.83—14.17) | 10.6(8.72—12.48) | 13(8.03—17.97) | 11.6(9.03—14.17) | 38.8(30.31—47.29) | 28.6(19.28—37.92) |
| **ResNet-101** | 9(7.48—10.52) | 11(7.60—14.40) | 10.4(6.51—14.29) | 11(8.09—13.91) | 27(16.87—37.13) | 28(19.81—36.19) |
| **GoogLeNet** | 11.6(10.18—13.02) | 12.4(10.98—13.82) | 8(6.48—9.52) | 8.4(7.72—9.08) | 17.4(12.24—22.56) | 25(18.98—31.02) |
| **SqueezeNet** | 20.4(13.40—27.40) | 27.6(21.61—33.59) | 17.8(14.47—21.13) | 14.2(10.99—17.41) | 24.8(17.70—31.90) | 20.8(16.74—24.86) |

**Supplementary Table S2. Mean training time (hours) per fold with 95% confidence interval in parentheses**

| | Make 3 sets, then augment validation | Make 3 sets | Make 3 sets, then augment training | Make 3 sets, then augment both training and validation | Separate test set, augment the rest, then make 2 other sets | Augment all before making 3 sets |
|---|---|---|---|---|---|---|
| **Inception-v3** | 4.56(3.17—5.95) | 3.31(2.70—3.91) | 30.17(19.22—41.12) | 28.48(22.48—34.47) | 96.11(74.26—117.97) | 70.72(49.13—92.30) |
| **ResNet-101** | 4.73(3.89—5.57) | 4.04(2.64—5.44) | 30.52(19.95—41.09) | 32.49(25.27—39.71) | 79.30(50.46—108.14) | 82.35(60.15—104.55) |
| **GoogLeNet** | 1.02(0.92—1.13) | 0.77(0.69—0.86) | 3.85(3.08—4.61) | 4.30(4.08—4.52) | 9.12(6.43—11.82) | 15.25(10.89—19.62) |
| **SqueezeNet** | 0.88(0.59—1.16) | 0.72(0.57—0.88) | 3.92(2.91—4.93) | 3.32(2.61—4.04) | 5.86(3.92—7.81) | 4.78(3.83—5.74) |

Supplementary Table S3. Validation accuracy[*] with binomial exact 95% confidence interval in parentheses

| | Make 3 sets, then augment validation | Make 3 sets | Make 3 sets, then augment training | Make 3 sets, then augment both training and validation | Separate test set, augment the rest, then make 2 other sets | Augment all before making 3 sets |
|---|---|---|---|---|---|---|
| **Inception-v3** | 92.85%(92.68%—93.01%) | 93.48%(93.02%—93.92%) | 96.67%(96.33%—96.98%) | 96.78%(96.66%—96.89%) | 99.67%(99.63%—99.71%) | 99.41%(99.36%—99.46%) |
| **ResNet-101** | 92.33%(92.15%—92.49%) | 94.63%(94.21%—95.03%) | 96.34%(95.99%—96.68%) | 96.36%(96.23%—96.47%) | 99.28%(99.23%—99.33%) | 98.94%(98.87%—99.00%) |
| **GoogLeNet** | 91.75%(91.57%—91.92%) | 93.42%(92.96%—93.86%) | 95.56%(95.17%—95.93%) | 95.78%(95.65%—95.91%) | 98.50%(98.42%—98.58%) | 98.51%(98.43%—98.59%) |
| **SqueezeNet** | 90.96%(90.78%—91.15%) | 93.36%(92.89%—93.80%) | 94.94%(94.53%—95.33%) | 94.86%(94.72%—95.00%) | 96.81%(96.70%—96.92%) | 96.33%(96.20%—96.44%) |

**[*]The accuracy is a weighted mean of the sensitivity and the specificity, weighted by the prevalence. Since the prevalence differs between the dataset and the population, values in this table are not representative of the population.**

**Supplementary Table S4. Testing accuracy* with binomial exact 95% confidence interval in parentheses**

| | Make 3 sets, then augment validation | Make 3 sets | Make 3 sets, then augment training | Make 3 sets, then augment both training and validation | Separate test set, augment the rest, then make 2 other sets | Augment all before making 3 sets |
|---|---|---|---|---|---|---|
| **Inception-v3** | | | | | | |
| • **Non-augmented test set** | 94.33% (93.89%—94.74%) | 93.67% (93.22%—94.11%) | 96.57% (96.23%—96.89%) | 96.79% (96.45%—97.10%) | 97.24% (96.93%—97.53%) | |
| • **Augmented test set** | 92.82% (92.66%—92.99%) | 92.33% (92.16%—92.50%) | 96.61% (96.49%—96.72%) | 96.65% (96.53%—96.76%) | 97.15% (97.04%—97.25%) | 99.38% (99.33%—99.43%) |
| **ResNet-101** | | | | | | |
| • **Non-augmented test set** | 94.29% (93.86%—94.71%) | 94.83% (94.41%—95.22%) | 96.16% (95.80%—96.50%) | 96.48% (96.13%—96.81%) | 96.76% (96.42%—97.07%) | |
| • **Augmented test set** | 92.34% (92.17%—92.51%) | 92.93% (92.77%—93.09%) | 96.14% (96.01%—96.26%) | 96.37% (96.24%—96.48%) | 96.70% (96.58%—96.81%) | 98.99% (98.93%—99.06%) |
| **GoogLeNet** | | | | | | |
| • **Non-augmented test set** | 93.39% (92.93%—93.83%) | 93.11% (92.64%—93.56%) | 95.33% (94.93%—95.71%) | 95.70% (95.31%—96.06%) | 95.99% (95.62%—96.33%) | |
| • **Augmented test set** | 91.92% (91.75%—92.10%) | 92.11% (91.93%—92.28%) | 95.44% (95.30%—95.57%) | 95.62% (95.49%—95.75%) | 95.92% (95.79%—96.04%) | 98.51% (98.43%—98.58%) |
| **SqueezeNet** | | | | | | |
| • **Non-augmented test set** | 91.63% (91.12%—92.13%) | 93.82% (93.37%—94.25%) | 95.40% (95.00%—95.77%) | 95.18% (94.78%—95.56%) | 95.39% (95.00%—95.76%) | |
| • **Augmented test set** | 91.28% (91.10%—91.46%) | 92.81% (92.65%—92.98%) | 95.42% (95.29%—95.56%) | 94.85% (94.70%—94.99%) | 95.36% (95.22%—95.49%) | 96.44% (96.32%—96.56%) |

***The accuracy is a weighted mean of the sensitivity and the specificity, weighted by the prevalence. Since the prevalence differs between the dataset and the population, values in this table are not representative of the population.**

Supplementary Table S5. Sensitivity (recall) with binomial exact 95% confidence interval in parentheses

| | Make 3 sets, then augment validation | Make 3 sets | Make 3 sets, then augment training | Make 3 sets, then augment both training and validation | Separate test set, augment the rest, then make 2 other sets | Augment all before making 3 sets |
|---|---|---|---|---|---|---|
| **Inception-v3** | | | | | | |
| • **Non-augmented test set** | 93.58% (92.92%—94.20%) | 94.99% (94.40%—95.54%) | 96.97% (96.50%—97.40%) | 97.11% (96.65%—97.52%) | 96.85% (96.37%—97.28%) | |
| • **Augmented test set** | 92.77% (92.53%—93.00%) | 94.64% (94.44%—94.85%) | 97.21% (97.05%—97.35%) | 97.00% (96.84%—97.15%) | 96.93% (96.77%—97.09%) | 99.38% (99.30%—99.45%) |
| **ResNet-101** | | | | | | |
| • **Non-augmented test set** | 93.10% (92.42%—93.74%) | 94.87% (94.27%—95.42%) | 95.22% (94.64%—95.75%) | 96.02% (95.49%—96.51%) | 96.61% (96.11%—97.06%) | |
| • **Augmented test set** | 90.25% (89.98%—90.52%) | 92.90% (92.66%—93.13%) | 95.27% (95.08%—95.46%) | 95.83% (95.65%—96.01%) | 96.49% (96.32%—96.65%) | 98.63% (98.52%—98.74%) |
| **GoogLeNet** | | | | | | |
| • **Non-augmented test set** | 94.01% (93.37%—94.61%) | 93.65% (92.99%—94.26%) | 94.73% (94.13%—95.29%) | 95.18% (94.60%—95.72%) | 95.03% (94.44%—95.57%) | |
| • **Augmented test set** | 92.43% (92.19%—92.67%) | 92.93% (92.69%—93.16%) | 94.86% (94.65%—95.06%) | 95.20% (95.00%—95.40%) | 95.08% (94.88%—95.28%) | 99.01% (98.92%—99.10%) |
| **SqueezeNet** | | | | | | |
| • **Non-augmented test set** | 91.21% (90.45%—91.92%) | 96.13% (95.60%—96.61%) | 94.46% (93.84%—95.03%) | 97.38% (96.94%—97.78%) | 96.95% (96.48%—97.38%) | |
| • **Augmented test set** | 91.35% (91.10%—91.61%) | 95.75% (95.56%—95.93%) | 94.41% (94.20%—94.62%) | 97.55% (97.41%—97.69%) | 97.21% (97.06%—97.36%) | 96.05% (95.87%—96.22%) |

**Supplementary Table S6. Specificity with binomial exact 95% confidence interval in parentheses**

| | Make 3 sets, then augment validation | Make 3 sets | Make 3 sets, then augment training | Make 3 sets, then augment both training and validation | Separate test set, augment the rest, then make 2 other sets | Augment all before making 3 sets |
|---|---|---|---|---|---|---|
| **Inception-v3** | | | | | | |
| • **Non-augmented test set** | 95.06% (94.48%—95.59%) | 92.38% (91.68%—93.05%) | 96.18% (95.67%—96.66%) | 96.47% (95.97%—96.92%) | 97.63% (97.21%—98.00%) | |
| • **Augmented test set** | 92.88% (92.65%—93.11%) | 90.07% (89.79%—90.33%) | 96.03% (95.85%—96.20%) | 96.31% (96.13%—96.48%) | 97.36% (97.21%—97.50%) | 99.38% (99.31%—99.45%) |
| **ResNet-101** | | | | | | |
| • **Non-augmented test set** | 95.46% (94.90%—95.98%) | 94.79% (94.19%—95.34%) | 97.09% (96.63%—97.50%) | 96.92% (96.45%—97.35%) | 96.91% (96.43%—97.33%) | |
| • **Augmented test set** | 94.39% (94.18%—94.60%) | 92.96% (92.73%—93.19%) | 96.99% (96.83%—97.14%) | 96.89% (96.73%—97.04%) | 96.90% (96.74%—97.06%) | 99.35% (99.27%—99.42%) |
| **GoogLeNet** | | | | | | |
| • **Non-augmented test set** | 92.79% (92.10%—93.43%) | 92.59% (91.89%—93.24%) | 95.91% (95.38%—96.40%) | 96.20% (95.68%—96.67%) | 96.92% (96.45%—97.35%) | |
| • **Augmented test set** | 91.43% (91.17%—91.68%) | 91.30% (91.04%—91.55%) | 96.00% (95.82%—96.18%) | 96.03% (95.85%—96.20%) | 96.74% (96.57%—96.90%) | 98.01% (97.88%—98.14%) |
| **SqueezeNet** | | | | | | |
| • **Non-augmented test set** | 92.05% (91.33%—92.72%) | 91.56% (90.82%—92.25%) | 96.32% (95.81%—96.78%) | 93.02% (92.35%—93.66%) | 93.86% (93.22%—94.46%) | |
| • **Augmented test set** | 91.21% (90.95%—91.46%) | 89.95% (89.67%—90.22%) | 96.41% (96.24%—96.57%) | 92.20% (91.96%—92.44%) | 93.54% (93.32%—93.76%) | 96.82% (96.66%—96.98%) |

**Supplementary Table S7. Area under the receiver operating characteristic curve with binomial exact 95% confidence interval in parentheses**

| | Make 3 sets, then augment validation | Make 3 sets | Make 3 sets, then augment training | Make 3 sets, then augment both training and validation | Separate test set, augment the rest, then make 2 other sets | Augment all before making 3 sets |
|---|---|---|---|---|---|---|
| **Inception-v3** | | | | | | |
| • **Non-augmented test set** | .9856 (.9833—.9877) | .9840 (.9816—.9862) | .9943 (.9927—.9955) | .9949 (.9934—.9961) | .9961 (.9948—.9972) | |
| • **Augmented test set** | .9798 (.9789—.9807) | .9790 (.9780—.9799) | .9943 (.9938—.9948) | .9949 (.9944—.9953) | .9959 (.9954—.9963) | .9997 (.9996—.9998) |
| **ResNet-101** | | | | | | |
| • **Non-augmented test set** | .9878 (.9856—.9897) | .9881 (.9860—.9900) | .9926 (.9909—.9941) | .9942 (.9927—.9955) | .9947 (.9932—.9959) | |
| • **Augmented test set** | .9780 (.9771—.9790) | .9803 (.9793—.9811) | .9926 (.9920—.9931) | .9941 (.9936—.9946) | .9945 (.9940—.9949) | .9994 (.9992—.9995) |
| **GoogLeNet** | | | | | | |
| • **Non-augmented test set** | .9826 (.9801—.9849) | .9833 (.9808—.9855) | .9915 (.9896—.9931) | .9919 (.9901—.9934) | .9927 (.9910—.9942) | |
| • **Augmented test set** | .9755 (.9745—.9765) | .9779 (.9770—.9789) | .9916 (.9910—.9922) | .9918 (.9912—.9924) | .9926 (.9921—.9932) | .9991 (.9988—.9992) |
| **SqueezeNet** | | | | | | |
| • **Non-augmented test set** | .9743 (.9713—.9771) | .9859 (.9836—.9879) | .9912 (.9893—.9928) | .9906 (.9887—.9923) | .9906 (.9887—.9923) | |
| • **Augmented test set** | .9714 (.9703—.9724) | .9825 (.9816—.9833) | .9912 (.9906—.9918) | .9905 (.9898—.9911) | .9909 (.9903—.9915) | .9949 (.9944—.9953) |
